# Extracorporeal Membrane Oxygenation as a Bridge for Heart Failure and Cardiogenic Shock

**DOI:** 10.1155/2016/7263187

**Published:** 2016-05-16

**Authors:** Zhao-peng Zhong, Hong Wang, Xiao-tong Hou

**Affiliations:** Center for Cardiac Intensive Care, Beijing Anzhen Hospital, Capital Medical University, Anzhen Road, Beijing 100029, China

## Abstract

Heart failure (HF) can be defined as cardiac structural or functional abnormality leading to a series of symptoms due to deficiency of oxygen delivery. In the clinical practice, acute heart failure (AHF) is usually performed as cardiogenic shock (CS), pulmonary edema, and single or double ventricle congestive heart failure. CS refers to depressed or insufficient cardiac output (CO) attributable to myocardial infarction, fulminant myocarditis, acute circulatory failure attributable to intractable arrhythmias or the exacerbation of chronic heart failure, postcardiotomy low CO syndrome, and so forth. Epidemiological studies have shown that CS has higher in-hospital mortality in patients with AHF. Besides, we call the induced, sustained circulatory failure even after administration of high doses of inotropes and vasopressors refractory cardiogenic shock. In handling these cases, mechanical circulatory support devices are usually needed. In this review, we discuss the current application status and clinical points in utilizing extracorporeal membrane oxygenation (ECMO).

## 1. Introduction

Extracorporeal membrane oxygenation (ECMO) evolved from cardiopulmonary bypass (CPB) and provides prolonged hemodynamic and respiratory support outside of the operating suite [1]. With the development of technology and safety, the use of ECMO has expanded, with increasing interest in patients with cardiogenic shock [2].

**Figure 1:**
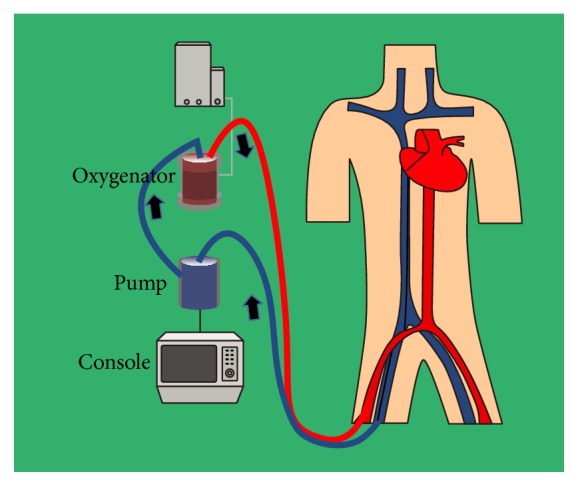
Venoarterial extracorporeal membrane oxygenation circuit via femoral arterial and venous cannulation.

The basic ECMO circuit includes a flow pump, an external membrane oxygenator, vascular cannulas, connecting tubes, a heat exchanger, and monitoring devices [3] (Figure 1). The circuit can be configured with two types: 2 venous cannulas (venovenous (VV) ECMO) or a venous and an arterial cannula (VA ECMO). In VV ECMO, blood is drained via an inflow cannula in vena cava and returned via an outflow cannula in right atrium. The hemodynamic stabilization depends on patients' intrinsic cardiac output (CO) in VV ECMO; hence, its application is for isolated respiratory failure. This modality was used successfully in acute respiratory distress syndrome (ARDS) during influenza A (H1N1) pandemic [4, 5]. In VA ECMO, blood is drained via a venous inflow cannula in the vena cava and returned via an outflow cannula to the arterial system. VA ECMO bypassing the heart and lungs depends on the driving force generating from pump instead of native CO. Therefore, VA ECMO provides short-term circulatory and respiratory support in patients with CS [6].

In this review, we focus on the current application status and clinical points of VA ECMO in CS.

## 2. VA ECMO

### 2.1. Indications

ECMO is a high-tech medical treatment option that requires a multidisciplinary team with specialists from cardiothoracic surgery, cardiology, perfusion, intensive care medicine, anesthesiology, respiratory care, and nursing care [7, 8]. The decision to institute ECMO should be based on the prior experience of ECMO team and hospital resources [9].

Etiological studies have shown that CS is mainly caused by myocardial infarction, fulminant myocarditis, the acute exacerbation of chronic heart failure (CHF), acute circulatory failure attributable to intractable arrhythmias, postcardiotomy low CO syndrome, and acute heart failure attributable to drug intoxication, and so forth [10–12]. Resulting in inadequate circulation despite conservative treatment (including volume load, inotropes, intra-aortic balloon counterpulsation (IABP), etc.), VA ECMO should be considered [13]. VA ECMO is not befitting of isolated respiratory failure. When CS appears concomitant with respiratory failure, VA ECMO performs more competitively over pure mechanical circulatory support devices [14], such as Impella and TandemHeart. VA ECMO may also be indicated in patients experiencing a cardiac arrest undergoing cardiopulmonary resuscitation [15], so called extracorporeal CPR (eCPR).

 Common indications for venoarterial extracorporeal membrane oxygenation (VA ECMO) are as follows:

- Cardiogenic shock:
  - Acute MI.
  - Fulminant myocarditis.
  - Acute exacerbation of CHF.
  - Acute circulatory failure attributable to intractable arrhythmias.
  - Postcardiotomy low CO syndrome.
  - Acute heart failure attributable to drug intoxication.
- Possible concomitant respiratory failure (concomitant respiratory failure is not necessary for indication of venoarterial membrane oxygenation but compels consideration of VA ECMO over other mechanical circulatory support devices):
  - Severe ARDS (defined as PaO_2_ : FiO_2_ is ≤100 mmHg on ventilators setting that includes positive end-expiratory pressure ≥5 cmH_2_O, with supportive clinical features including compliance <0.5 mL/cmH_2_O/kg).
  - Severe, refractory hypoxia (PaO_2_ : FiO_2_ ratio < 100).
  - Hypercapnic respiratory failure (arterial PH < 7.20).
  - Significant or symptomatic pulmonary hypertension.
  - Pulmonary shunt fraction >30%.
- Cardiac arrest requiring eCPR.

### 2.2. Contraindications

In general, ECMO may be regarded as a bridge to anticipated recovery, to heart or lung transplant, or to long-term ventricular assist device (VAD). Hence, the most common contraindication is nonrecoverable cardiac failure without indication for transplant or long-term VAD. ECMO should not be instituted to increase the course and costs of the illness without any subsistent possibility of survival or acceptable quality of life [16]. ECMO may also be contraindicated in patients with severe brain damage due to insufficient perfusion especially in situations with delayed or noneffective resuscitation [17].

Other specific contraindications include continuously progressive systemic disease (such as malignancy or chronic obstructive pulmonary disease (COPD), etc.) [18], coagulation disorder (including active bleeding, certain recent surgeries, or hemorrhagic intracranial event), existent multiorgan failure, and dissatisfied correction of cardiac deformity. VA ECMO is also contraindicated in patients with severe aortic regurgitation or aortic dissection.

 Common contraindications to venoarterial extracorporeal membrane oxygenation (VA ECMO) are as follows:

- Absolute contraindications:
  - Nonrecoverable cardiac failure without indication for transplantation or long-term VAD.
  - Severe, irreversible brain damage without realistic possibility of reviving.
  - Severe aortic regurgitation.
- Relative contraindications:
  - Continuously progressive systemic diseases.
  - Coagulation disorder.
  - Existent multiorgan failure.
  - Dissatisfied correction of cardiac deformity.
  - Mechanical ventilation >10 days.
  - Aortic dissection.

### 2.3. Implantation

The circuit should be proper flushed with multiple electrolyte solution or normal saline priming. Cannulation for VA ECMO can be central or peripheral. In central cannulation, a venous cannula is inserted in the right atrium and an arterial cannula is implanted in the ascending aorta. This mode is mainly chosen in compelled implantation with open-heart surgery. In peripheral cannulation, venous cannula from femoral vein to the inferior vena cava and femoral arterial cannulation (IVC-FA) are mostly adopted for VA ECMO in adults [19]. Besides, the internal jugular vein and the axillary artery or common carotid artery can be considered in special occasions. The right internal jugular vein and common carotid artery are appropriate in infants since their femoral vessels are not well developed. Access for cannulation can be percutaneous or surgical. The Seldinger technique may reduce procedure time and surgical trauma. Surgical access provides a thorough visual inspection for placement. Distal limb ischemia can be significantly avoided by insertion of an anterograde perfusion cannula (usually 6–8 Fr) into the primary distal artery [6].

### 2.4. Management

Available management of ECMO needs in-depth knowledge and controls of clinical targets related to extracorporeal circulation. After anticoagulation with unfractionated heparin for an activated clotting time (ACT) of 180–210 seconds or an activated partial thromboplastin time (APTT) of >1.5 times the normal, the ECMO assist can be undertaken. Initial settings are shown as follows: perfusion flow of 50–80 mL/kg·min in adults (80–150 mL/kg·min in children and infants); arterial oxyhemoglobin saturation (SaO_2_) >90% (measured in the patient's right radial arterial); venous oxyhemoglobin saturation (SvO_2_) >65% (measured in venous drainage tube); hematocrit of 30–40% or with hemoglobin (Hb) >10 mg/dL or more; blood platelet count >10 000/mm^3^ [19]. Gas flow rate or oxygen concentration can be increased to ensure 100% fraction of inspired oxygen (FiO_2_) in the arterial line and partial pressure of oxygen (PaO_2_) of 100–150 mmHg in patient's right radial artery. Increased gas flow can realize CO_2_ clearance and reduce arterial partial pressure of CO_2_ (PaCO_2_) which is usually controlled between 35 and 45 mmHg. Mild hypothermia may be recommended if brain damage is suspected. Fever and chill should be treated to decrease oxygen consumption [21]. Oxygen consumption (VO_2_) can be calculated according to the following formula:

(1) $$\begin{array}{c} {VO}_{2}=\left(\;{SaO}_{2}-{SvO}_{2}\;\right)\times Hb\times 1.36\times F, \end{array}$$

where VO_2_ is oxygen consumption (mL/min), SaO_2_ is arterial oxyhemoglobin saturation (%), SvO_2_ is venous oxyhemoglobin saturation (%), Hb is hemoglobin (mg/dL), and F is blood flow rate (L/min).

### 2.5. Weaning

Pulsatility in arterial waveform and decrease of the central venous pressure mean recovery of ventricular systolic function, which can be intuitively seen by echocardiogram. In addition, improvements in SaO_2_, pulmonary compliance measures, and chest X-ray can suggest respiratory recovery [6, 14]. Once there is durable evidence of cardiac and respiratory recovery, trails of discontinuation may be undertaken. Usually, in support of moderate-dose positive inotropic agents, the circuit flow can be decreased to 1-2 L/min (or 20–30 mL/kg·min) or even lower. Another way to discontinuation of VA ECMO is tubing an external bridge between the arterial and venous lines, which allows blood to circulate continually when clamping the arterial and venous cannulas [6]. Such trails with high risk of thrombosis need an increase in heparin dosage and should be shortened as possible [20].

### 2.6. Complications

Directly fatal complications of VA ECMO include the circuit rupture and plenty of gas embolism. The most common complications are bleeding and thrombosis. Potential bleeding sites may be cannulas or surgical, gastrointestinal, cerebral, or tracheotomy incision, with a total incidence of 34%. Thrombosis (up to 17%) mostly develops in the arterial cannula and oxygenator [2]. When visible thrombus exists, or with poor arterial oxygenation, certain parts of the circuit can be renewed. Thrombosis may also occur by blood stasis in left ventricle or aortic root due to severe left ventricle dysfunction or retrograde antagonistic flow [20]. More distal limb ischemia may be avoided by insertion of an anterograde distal-perfusion cannula; still 6% of VA ECMO patients need decompression for osteofascial compartment syndrome. Other common complications include hemolysis, intravascular coagulation, stroke, and intubated vessel stenosis [22].

## 3. Clinical Views

It is well known that CS could manifest as depressed or insufficient cardiac output, presenting a reduced cardiac index and low blood pressure which induces poor myocardial perfusion and profound depression of myocardial contractility [23]. The reduction in cardiac index could lead to severe tissue hypoperfusion which is most sensitively monitored by serum lactate and may finally cause death if the circulation is not timely rebuilt [24]. In current guidelines, early revascularization by either percutaneous intervention (PCI) or coronary artery bypass grafting (CABG) is a class 1B recommendation [25]. However, rates of 50–70% in registries are still unsatisfactory even though the application of early revascularization has greatly increased in clinical practice [11, 26]. Besides, the basic treatment measures include volume expansion, vasopressors, and inotropes plus additional therapy for the prevention or treatment of multiorgan dysfunction system (MODS). Despite the stabilization of hemodynamics in CS, there are few randomized data showing a prognostic benefit and significant reduction in mortality. To overcome the limitations of inotropes and vasopressors to maintain adequate perfusion pressure and improve outcome, mechanical circulatory support became appealing. Previous reviews have shown the evidence of mechanical circulatory support in CS [14, 16]. Therefore, only major clinical points of view are covered here.

Protective lung ventilation strategies should be undertaken to minimize ventilator-induced lung injury, such as barotrauma and volutrauma, and accelerate respiratory recovery as possible. These include using positive end-expiratory pressure (PEEP) of usually 6–10 cmH_2_O to keep alveolar recruitment, limiting tidal volumes (VT) to 4–6 cc/kg, breathing rate to 4–8 times/min, FiO_2_ to no more than 50%, and airway plateau pressure to ≤30 cmH_2_O. If the SvO_2_ is below the target, blood flow rate can be increased. Expansion of intravascular volume and increasing of hemoglobin concentration may also be undertaken to ensure adequate tissue perfusion. Majority of CO_2_ removal can be accomplished by an increasing of gas flow to oxygenator, instead of ventilator setting. Changes in flow rate on hemodynamics should be closely supervised with monitoring of ventilator settings.

Decompression of the right and left ventricle should be achieved in patients with VA ECMO because myocardial injury may occur if atrium and ventricle become distended. Frequent echocardiograms are recommended to evaluate cardiac function. Depression of the right ventricle can be realized by increasing drainage flow and adjusting the position of venous cannula. Adequate venous drainage is the key point to prevent excessive preload. Still, a small amount of blood may pass through pulmonary circulation, causing left heart overexpansion when lacking enough contractility to open the aortic valve. In this situation, addition of a vent cannula or percutaneous VAD, such as Impella, should be considered to decompress the ventricle [27].

**Figure 2:**
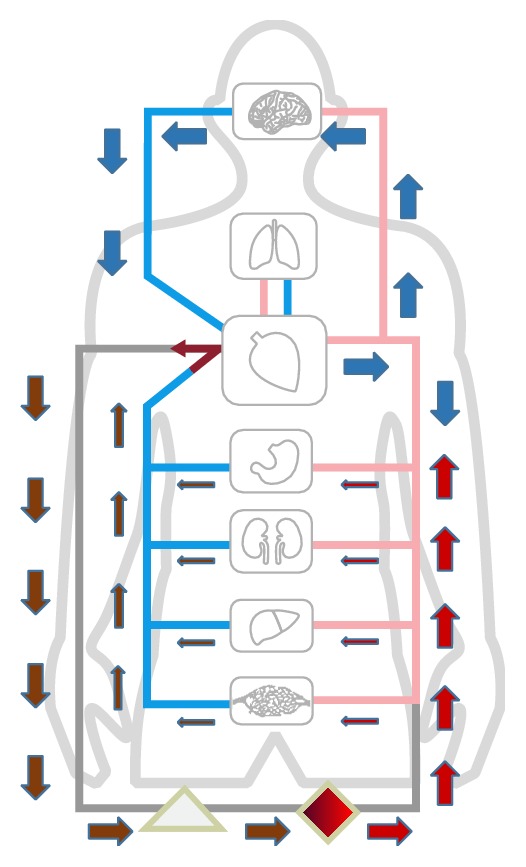
Differential hypoxia formed by opposed flow between retrograde oxygenated flow and anterograde deoxygenated flow ejected by native CO. The lower body was perfused by extracorporeal oxygenated blood, whereas the upper body was perfused by deoxygenated flow from the pulmonary circulation when concomitant with respiratory failure. Limited by femoral venous cannula, high SO_2_ blood from the inferior vena cava was drained back to the ECMO; blood from the superior vena cava with lower SO_2_ could not be completely drained. Nondrainage venous blood ejected by native CO continually filled the upper body and coronary artery that dual circulation occurred.

Differential hypoxia has been reported which could cause insufficient oxygen supply to vital organs, such as the brain and heart, in respiratory dysfunction patients with femoral VA ECMO (from the inferior vena cava to the femoral artery, IVC-FA) [28]. If the femoral artery cannulation is chosen, oxygenated blood flow will retrograde up the descending aorta and into ascending aorta, to supply the coronary arteries and cerebral vessels. When concomitant with respiratory failure, anterograde deoxygenated flow ejected by native CO may form a mixing zone with retrograde oxygenated flow from the femoral arterial cannula. As native cardiac function recovers, the mixing zone may move along with aorta that hypoxia of myocardium and upper body will occur (Figure 2). Monitoring pulse oxygen saturation or blood gas analysis in right radial artery will suggest whether adequate cerebral oxygen is provided. As hypoxia occurs in the upper body, dual circulation was proposed as the major reason. Oxygenated blood from the ECMO circuit enters the descending aorta to perfuse the lower body, whereas the inadequate oxygenated blood flow of upper body is from the left ventricle. To ameliorate this phenomenon, some clinicians have suggested (1) modifying FA cannulation to the axillary artery (IVC-AA) or common carotid artery (IVC-CA) or (2) using venoarteriovenous ECMO by adding an additional venous reinfusion cannula in the internal jugular vein to IVC-FA (FA-IJV) [29]. Besides, Kitamura and colleagues reported that differential hypoxia could be ameliorated when IVC-FA was modified with superior vena cava (SVC) drainage (SVC-FA), which directly delivers oxygenated blood to upper body [30]. Animal models had been made to mimic differential hypoxia in VA ECMO as determined by SvO_2_ and a significant difference was found between the IVC and the SVC, explaining how differential hypoxia is attenuated by alternative modes of cannulation [31].

Neurological morbidity has become a significant concern as a more important role played by VA ECMO in treating acute heart failure for many patients. Brain death occurred in 7% to 21% of the cases of ECMO treated adults in some ECMO centers [32]. Additionally, approximately one-half of the survival patients showed evidence of cerebral injury. Cerebral blood flow (CBF) reduction and nonpulsating blood flow during VA ECMO might play a role in the pathogenesis of this complication. From another aspect, the addition of an intra-aortic balloon pump (IABP) during peripheral VA ECMO support has been shown to improve coronary bypass graft flows and cardiac function in refractory cardiogenic shock after cardiac surgery. Some studies had shown the additional IABP support on the CBF in patients with peripheral VA ECMO following cardiac procedures and found changes to CBF depending on the anterograde flow by spontaneous cardiac function [33]. The addition of an IABP to VA ECMO support significantly decreased the mean of the CBF during myocardial stunning but increased the mean of the CBF during the recovery of cardiac function. The pulsatility effect of IABP probably leads to improving CBF perfusion and contributes to cerebral autoregulation recovery. Besides, combined application of IABP has a potential indication in preventing hydrostatic pulmonary edema during peripheral VA ECMO. A retrospective cohort of 259 peripheral VA ECMO patients shows that the IABP group has a significantly lower radiologic score than the non-IABP group within seven days. Bréchot and his colleagues explained their study that IABP could partially unload the left ventricular and therefore reduce the risk of pulmonary edema related to the increase in left ventricular afterload induced by VA ECMO [34].

Lactate has been proposed as a marker of tissue perfusion that is influenced by not only macrocirculation but also microcirculation, whereas traditional hemodynamic parameters have been suggested to be unreliable [35]. Lactate has been proven to be associated with increased risks of death in infection, sepsis, trauma, and operations, including cardiac surgery. Clinical trials had shown the association between the dynamic behavior of lactate and mortality in postcardiotomy patients under ECMO support [36]. A randomized controlled trial included 123 adult patients who had undergone cardiac surgery and received VA ECMO implantation to treat refractory postcardiotomy cardiogenic shock [37]. A total of 56% of the patients were successfully weaned from ECMO support. The in-hospital mortality was 65.9% overall, similar to the data of 64% from the multicenter extracorporeal life support organization (ELSO) registry. Univariate and multivariate analyses indicated that age, gender, mean lactate concentration, and lactate clearance were reliable predictors of in-hospital mortality. The mean lactate concentration and lactate clearance 12 hours after the initiation of ECMO support provided better prognostic guidance. The mean lactate concentration and lactate clearance were able to predict successful weaning from ECMO in the 12-hour model only. To predict survival after ECMO for refractory cardiogenic shock, Schmidt and colleagues have established the survival after venoarterial ECMO- (SAVE-) score. Cases of 3846 patients with cardiogenic shock treated with ECMO from centers of international extracorporeal life support organization registry were analyzed and showed chronic renal failure, longer duration of ventilation prior to ECMO initiation, pre-ECMO organ failures, pre-ECMO cardiac arrest, congenital heart disease, lower pulse pressure, and lower serum bicarbonate (HCO_3_) were risk factors associated with mortality, whereas younger age, lower weight, acute myocarditis, heart transplant, refractory ventricular tachycardia or fibrillation, higher diastolic blood pressure, and lower peak inspiratory pressure were protective [38].

Several open questions remain in VA ECMO therapy such as insertion timing and appropriate patient selection. An early use of VA ECMO in CS patients could warrant the body perfusion and prevent the development of MODS. However, complications associated with invasive mechanical support devices might lead to adverse clinical outcome in patients who still had noninvasive therapeutic options. The balance between circulatory support efficacy and its device-related complications should be considered. Furthermore, there are still no widely approved, evidential, accurate criteria of initiating VA ECMO that the current decision is more subjective. Despite these uncertainties, considering the use of a percutaneous assist device for circulatory support in refractory CS is recommended by European and American guidelines.

## 4. Conclusions

ECMO is a high-tech medical treatment option that requires a multidisciplinary team. The decision to institute ECMO should be based on prior experience of ECMO team and hospital resources. VA ECMO plays a potential role in treating refractory cardiogenic shock, especially in patients with severe cardiogenic shock and combined respiratory failure. VA ECMO should be regarded as a bridge to anticipated recovery, to heart or lung transplant, or to long-term VAD. From the early days of VA ECMO support for refractory cardiogenic shock, the modality had been proved effective to prevent or reverse MODS, to improve hemodynamics and outcome in CS.

## Abbreviations

MI: Myocardial infarction
CHF: Chronic heart failure
CO: Cardiac output
ARDS: Acute respiratory distress syndrome
eCPR: Extracorporeal cardiopulmonary resuscitation
VAD: Ventricular assist device.
